# The development and validation of a cerebral ultrasound scoring system for infants with hypoxic-ischaemic encephalopathy

**DOI:** 10.1038/s41390-020-0782-0

**Published:** 2020-03-26

**Authors:** Kim V. Annink, Linda S. de Vries, Floris Groenendaal, Daniel C. Vijlbrief, Lauren C. Weeke, Charles C. Roehr, Maarten Lequin, Irwin Reiss, Paul Govaert, Manon J. N. L. Benders, Jeroen Dudink

**Affiliations:** 1 Department of Neonatology, Wilhelmina Children’s Hospital, University Medical Centre Utrecht, Utrecht University, Utrecht, the Netherlands; 2 Brain Centre Rudolf Magnus, University Medical Centre Utrecht, Utrecht University, Utrecht, the Netherlands; 3 Newborn Services, John Radcliffe Hospital, Oxford University Hospitals NHS Foundation Trust, Oxford, UK; 4 Department of Paediatrics, Medical Sciences Division, University of Oxford, Oxford, UK; 5 Department of Radiology, University Medical Centre Utrecht, Utrecht University, Utrecht, the Netherlands; 6 Department of Neonatology, Sophia Children’s Hospital, Erasmus University Hospital, Rotterdam, the Netherlands

## Abstract

**Background:**

Hypoxic-ischaemic encephalopathy (HIE) is an important cause of morbidity and mortality in neonates. When the gold standard MRI is not feasible, cerebral ultrasound (CUS) might offer an alternative. In this study, the association between a novel CUS scoring system and neurodevelopmental outcome in neonates with HIE was assessed.

**Methods:**

(Near-)term infants with HIE and therapeutic hypothermia, a CUS on day 1 and day 3–7 after birth and available outcome data were retrospectively included in cohort I. CUS findings on day 1 and day 3–7 were related to adverse outcome in univariate and the CUS of day 3–7 also in multivariable logistic regression analyses. The resistance index, the sum of deep grey matter and of white matter involvement were included in multivariable logistic regression analyses. A comparable cohort from another hospital was used for validation (cohort II).

**Results:**

Eighty-three infants were included in cohort I and 35 in cohort II. The final CUS scoring system contained the sum of white matter (OR = 2.6, 95% CI 1.5–4.7) and deep grey matter involvement (OR = 2.7, 95% CI 1.7–4.4). The CUS scoring system performed well in cohort I (AUC = 0.90) and II (AUC = 0.89).

**Conclusion:**

This validated CUS scoring system is associated with neurodevelopmental outcome in neonates with HIE.

## Introduction

Hypoxic-ischaemic encephalopathy (HIE) following presumed perinatal asphyxia is an important cause of morbidity and mortality in neonates and can result in long-term neurological sequelae.^1,2^ Perinatal asphyxia can be caused by acute or subacute perinatal hypoxia-ischaemia that both correspond with different patterns of brain injury.^3,4^ Acute perinatal asphyxia often results in injury of the deep grey nuclei, such as the basal ganglia and thalamus, or even in near-total brain injury.^5^ Injury to deep grey nuclei can lead to dyskinetic cerebral palsy, impaired cognitive outcome and epilepsy.^5^ Subacute (“chronic”) perinatal asphyxia is most often associated with watershed injury with involvement of the cortex and subcortical white matter.^5^ This usually does not result in motor impairment, but cognitive impairment and language problems occur more frequently and disabilities become apparent in childhood.^6–8^ In daily practice, the neurological prognosis is predicted based on the triad of neuroimaging, (amplitude-integrated) electro-encephalography ((a)EEG) and clinical features.^9^ Currently, the gold standard in neuroimaging is magnetic resonance imaging (MRI).^5,10,11^ MRI predicts neurological outcome in HIE based on conventional imaging i.e. with an MR scoring system^12,13^ and quantitatively with apparent diffusion coefficients, arterial spin labelling or magnetic resonance spectroscopy.^14–16^

MRI is the gold standard, but an alternative neuroimaging technique is necessary because there are circumstances when the infant is not stable enough to be transported to the MRI unit or MRI is not available, for example in developing countries.^17,18^ In these situations, cerebral ultrasound (CUS) might offer a bedside and cheaper alternative.^18^ Currently, CUS is not routinely used to predict outcome in HIE; CUS is not as sensitive as MRI in diagnosing brain injury and may take several days to become apparent.^11,19^ However, based on the available literature, CUS might not only be complementary to MRI but in some cases the only available neuroimaging method in HIE.^19–21^

A validated composite CUS scoring system is needed to assess brain injury and to predict outcome with CUS in HIE. To the best of our knowledge, such an ultrasound scoring system is not yet available. The CUS scoring systems that have previously been developed have not been validated.^17,22–28^ The aim of this study is to assess the association between a novel CUS scoring system and neurodevelopmental outcome in neonates with HIE at the age of 2 years.

## Methods

### Study population

We included (near-) term infants (36.0 until 42.0 weeks of gestational age) with HIE who were treated with hypothermia in the University Medical Centre Utrecht between January 2008 and July 2014, who had at least one CUS on day 1 and a second CUS between days 3 and 7 after birth. An additional inclusion criterion was the availability of outcome data, either death or a neurodevelopmental follow-up examination at the age of 2 years. We excluded infants with metabolic or genetic abnormalities. We used this cohort to create the scoring system (cohort I).

A different cohort with similar clinical characteristics, born in the Erasmus Medical Centre in Rotterdam, with at least one CUS between days 3 and 7 and available outcome data were used to validate the scoring system (cohort II). The same inclusion and exclusion criteria applied to this cohort. In both cohorts the CUS scans were part of standard clinical care. The scans were conducted by trained neonatologists, fellows in neonatology and physician assistants with different levels of experience in CUS. In cohort I CUS were performed using an ultrasound machine from Toshiba (Medical System Corporation, Tokyo, Japan) and in cohort II from Esaote (Genova, Italy). Convex 5–10 MHz and linear 15–18 MHz probes were used in both cohorts.

### Development of the CUS scoring system

**Fig. 1:**
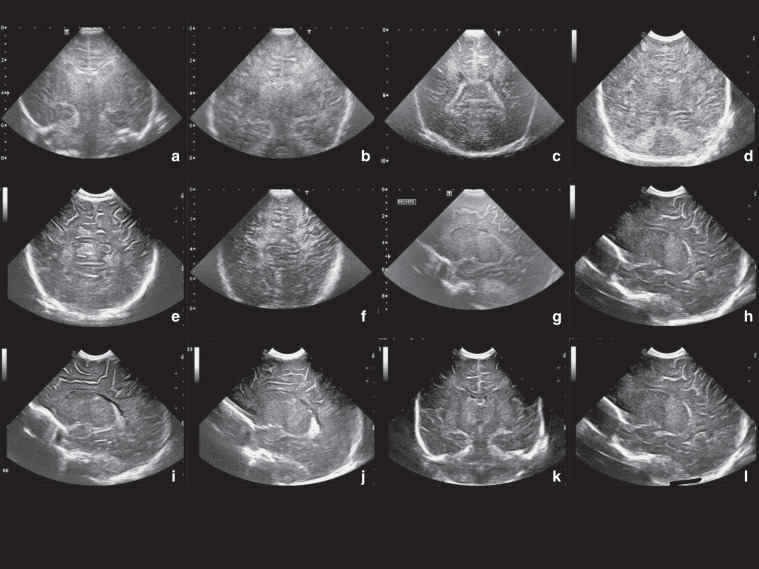
Cerebral ultrasound, outcome and HIE: example images. **a** Moderate cerebral oedema (1 point), **b** severe cerebral oedema (2 points), **c** moderate periventricular white matter (1 point), **d** severe periventricular white matter (2 points), **e** moderate subcortical white matter (1 point), **f** severe subcortical white matter (2 points), **g** moderate thalamus (1 point), **h** severe thalamus (2 points), **i** moderate putamen (1 point), **j** severe putamen (2 points), **k** “four-column sign” which means that both left and right thalamus and putamen are visible at the coronal view as four columns (1 point), **l** visibility of the PLIC (1 point). For the scoring sheet and definitions, see Table 1.

We searched the literature for possible CUS items to include in the scoring system. The relevance of all these items was discussed by a group of experts including neonatologists and a paediatric neuroradiologist, all with many years of experience in CUS (J.D., L.S.d.V., F.G., P.G., M.L.). Items were categorised into normal-mild, moderate and severe or into absent and present. The same group of experts reached consensus on the definitions of the different categories. For examples of the items see Fig. 1 and for definitions see Table 1. Additional antenatally acquired pathology was also scored, i.e. porencephaly and atrophy.

**Table 1:** The scoring system.

Item	Normal-mildly abnormal (0)	Moderately abnormal (1)	Severely abnormal (2)	Total points
Impaired white/grey matter differentiation and/or slit-like ventricles	Normal differentiation between grey and white matter and open ventricles.	Reduced differentiation between grey and white matter and/or slit-like ventricles.	No differentiation between grey and white matter and slit-like ventricles.	
Hyperechogenicity periventricular white matter	Normal echogenicity or minor hyperechogenicity.	Moderate or focal hyperechogenicity, not as white as choroid plexus.	Severe and diffuse hyperechogenicity, as white as choroid plexus.	
Hyperechogenicity subcortical white matter	Normal echogenicity or minor hyperechogenicity.	Focal hyperechogenicity of the subcortical white matter. Moderate differentiation of white and (subcortical) grey matter.	Clear “tramlines” sign; hyperechogenicity of subcortical white matter almost similar to sulci with hyposignal intensity of cortex in between.	
Hyperechogenicity thalamus	Normal echogenicity or minor hyperechogenicity.	Moderate or focal hyperechogenicity thalamus.	The hyperechogenicity is severe and diffuse.	
Hyperechogenicity putamen	Normal echogenicity or minor hyperechogenicity.	Moderate or focal hyperechogenicity putamen	The hyperechogenicity is severe and diffuse.	

	Absent (0)	Present (1)		Total points
Four column sign	Normal echogenicity to minor hyperechogenicity	On the coronal cUS plane there is a four column sign caused by moderate or severe bilateral hyperechogenicity of the thalamus and putamen.		
Visibility PLIC	The PLIC is not visible as a hypo-echogenic line between the putamen and thalamus	The PLIC is clearly visible as a hypo-echogenic line between the hyperechogenic putamen and thalamus		

White matter involvement is the sum of oedema, periventricular and subcortical white matter damage (0–6 points). Grey matter involvement includes hyperechogenicity of the thalami, putamen, visibility of the PLIC, and four-column sign (0–6 points).

After the development of the scoring system, a neonatologist, with more than 10 years of experience in neonatal neurology and CUS, scored all CUS in cohort I (observer 1). First, the association between all separate items and adverse outcome was determined. Because of multicollinearity all white matter items were summed into a white matter score, the deep grey matter items into a grey matter score and the resistance index remained a separate item. Next, the association between the different composite scores in the scoring system and adverse outcome was calculated.

Furthermore, the additional value of day 1 CUS to diagnose antenatally acquired pathology of the brain was determined.

### Neurodevelopmental outcome

We retrospectively collected clinical parameters of all infants. Neurodevelopmental follow-up was performed with the Bayley Scales of Infant and Toddler Development, third edition (BSITD-III) at the age of 2 years^29^ by a neonatologist and an educational therapist or child psychologist. Adverse outcome was defined as death, cerebral palsy or a cognitive/motor composite score <85 according to the BSITD-III (United States of America norms) at 2 years of age.

### Validation: inter-observer variability

A neonatologist (J.D.) and a paediatric neuroradiologist (M.L.), both with an expertise in CUS, scored the day 1 and day 3−7 CUS of cohort I independently of each other (observer 1 and observer 3). Observers 1 and 3 did not work in the UMC Utrecht in the period that the CUS were conducted, so they were completely blinded to outcome. Another neonatologist (D.V.) without a special focus on CUS scored the CUS of 20 randomly selected patients to determine the inter-observer agreement in daily clinical practice (observer 2).

### Validation of the scoring system in cohort II

The CUS of cohort II were scored by two neonatologists with more than 25 years of experience in reading cerebral ultrasound scans (F.G. and L.S.d.V.). They scored the CUS together and reached consensus about the CUS score of all infants. The observers did not work in the Erasmus Medical Centre at the moment the CUS were performed, so they were completely blinded to outcome.

### Validation: correlation with MRI and histology

Secondary outcomes were the correlation with MRI and histological findings. A correlation between the CUS scoring system and MRI was assessed using the MRI scoring system of Weeke et al.^13^ in cohort I. In cohort II the diffusion weighted sequences were often of suboptimal quality; therefore, the secondary outcomes were only analysed for cohort I.

### Statistical analyses

SPSS Version 21 was used for statistical analysis (IBM Corp., Armonk NY, USA). Differences in baseline characteristics between the two cohorts were calculated using the independent *t* test or Mann−Whitney *U* test for continuous variables and the *χ*^2^ test for categorical variables. Univariate logistic regression was performed with the CUS items as independent variables and outcome as dependent variable. Non-significant items were excluded from further analysis. The sum of white matter involvement, of deep grey matter involvement and a Doppler ultrasound resistance index (RI) of a cerebral artery ≤0.55 were calculated and included in backward multivariable logistic regression analysis. Variables with a *p* value < 0.05 were entered in the model and those with a *p* value ≥ 0.1 deleted. The inter-observer variability and the correlation with MRI were calculated using Spearman rank correlation test. Predictive values and ROC curves were determined for cohorts I and II per cut-off value. A *p* value < 0.05 was considered statistically significant.

## Results

### Study population

Between January 2008 and July 2014, 145 infants with HIE were treated with hypothermia in the University Medical Centre Utrecht. In total, 83 infants were included in cohort I. Infants were excluded because of genetic or congenital abnormalities (*n* = 9), preterm birth < 36 weeks (*n* = 12), because one or both of the CUS were not present (*n* = 27), only a few images of an examination were saved (*n* = 2), the quality was insufficient because of suboptimal settings (*n* = 6) or because of missing outcome data (*n* = 6).

In the Erasmus Medical Centre, 69 infants with HIE were treated with hypothermia in this period and 35 newborns were included in cohort II. Infants were excluded because of genetic or congenital abnormalities (*n* = 9), preterm birth < 36 weeks (*n* = 2), diagnosis of arterial ischaemic stroke (*n* = 2), because the CUS between days 3 and 7 was not present (*n* = 15) or because no follow-up data were available (*n* = 6).

The incidence of death due to redirection of care between infants who were included and infants who were excluded from the study did not differ significantly.

Baseline characteristics were comparable between the two cohorts except for Apgar scores (Table 2). The mean Apgar scores were lower in Cohort II. Four infants had a postnatal collapse within 2 h after birth, which explains their high Apgar scores. The reason for incomplete hypothermia in all infants was early rewarming because of redirection of care. The reason of death in both cohorts was redirection of care based on a poor neurological prognosis.

**Table 2:** Baseline characteristics.

Patient characteristic	Cohort I (*n* = 83)	Cohort II (*n* = 35)	*p* value
Male*, n* (%)	50 (60.2)	19 (54.3)	0.55
Gestational age, median weeks^+days^ (range)	40^+1^ (36^+0^–42^+4^)	39^+6^ (36^+3^–41^+6^)	0.18
Birth weight, median in gram (range)	3500 (2260–5000)	3425 (1780–4440)	0.36
Mode of delivery			0.07
Emergency caesarian section, *n* (%)	46 (55.4)	20 (57.1)	
Vaginal delivery, *n* (%)	25 (30.1)	6 (17.1)	
Vacuum extraction, *n* (%)	12 (14.5)	9 (25.7)	
Apgar score at 5 min, median (range)	3 (0–10)	3 (0–9)	<0.001
First pH, median (range)	6.96 (6.53–7.34)	6.94 (6.60–7.28)	0.67
Thompson score, median (range)	10 (4–19)	11 (5–20)	0.83
Sarnat classification			0.87
Mild, *n* (%)	5 (6.0)	5 (14.3)	
Moderate, *n* (%)	58 (69.9)	19 (54.3)	
Severe, *n* (%)	20 (24.1)	10 (28.6)	
Incomplete hypothermia <72 h, *n* (%)	6 (7.2)	0 (0)	0.10
Postmenstrual age at CUS day 3–7, median days (range)	4 (3–7)	4 (3–7)	0.49
MRI available, *n* (%)	77 (92.8)	N/A	N/A
Postmenstrual age at MRI, median days (range)	6 (3–16)	N/A	N/A
Outcome			0.26
Normal, *n* (%)	54 (65.1)	17 (48.6)	
Adverse outcome <85, on BSITD-III, *n* (%)	3 (3.6)	7 (20.0)	
Death, *n* (%)	26 (31.3)	11 (31.4)	

### Development of the CUS scoring system

The following potential CUS items were found in the literature: slit-like ventricles, impaired differentiation white/grey matter, “four-column” sign, hyperechogenicity of the thalamus, putamen, subcortical white matter, periventricular white matter, hippocampus, brainstem and vermis, visibility of the posterior limb internal capsule (PLIC) and RI of a cerebral artery ≤0.55.^17,22–28,30^ We excluded hyperechogenicity of the hippocampus, vermis and brainstem because these structures were rarely depicted on the available CUS images. The RI was scored based on all available ultrasounds; if the RI was ≤0.55 at one of these CUS, this item was scored as abnormal.

### The CUS scoring system and neurodevelopmental outcome on day 1

Antenatally acquired pathology was found in 14 infants (17%). Germinal layer cysts (*n* = 7), lenticulostriate vasculopathy (*n* = 4), frontal horn cysts (*n* = 2) and porencephaly (*n* = 1) were identified.

On day 1, only severe hyperechogenicity of the periventricular white matter on CUS was significantly associated with adverse outcome (OR = 5.0; 95% CI 1.4–18.4) in univariate logistic regression. The other items were not significantly associated with adverse outcome. Although it did not reach significance, all infants with hyperechogenicity of the thalamus (moderate *n* = 5; severe *n* = 1), of the putamen (moderate *n* = 2) or a four-column sign (*n* = 1) on day 1 had an adverse outcome.

### The CUS scoring system and neurodevelopmental outcome between days 3 and 7

Between days 3 and 7 after birth, most of the CUS items significantly predicted adverse outcome in the univariate logistic regression analysis (Table 3).

**Table 3:** Univariate association of the items on CUS (day 3–7) and adverse outcome.

Item	Category	Patients per category, *n*	OR (95% CI)
Cerebral oedema	Normal-mild	70	–
	Moderate	12	14.4 (2.9–72.3)
	Severe	1	
Thalamus	Normal-mild	45	–
	Moderate	32	26.7 (6.7–106.2)
	Severe	5	
Putamen	Normal-mild	66	–
	Moderate	15	7.9 (2.2–28.2)
	Severe	1	
Four-column sign	Normal	67	–
	Abnormal	14	10.0 (2.5–39.9)
PLIC	Normal	59	–
	Abnormal	23	11.1 (3.6–34.2)
Periventricular white matter	Normal-mild	32	–
	Moderate	43	9.2 (2.4–34.9)
	Severe	8	16.1 (2.5–103.6)
Subcortical white matter	Normal-mild	42	–
	Moderate	35	4.0 (1.5–11.1)
	Severe	6	8.5 (1.3–54.8)
Resistance index	Normal	60	–
	Abnormal	8	16.3 (1.9–142.6)

In the multivariable analysis the RI (0−1 point), the sum of the deep grey matter (0–6 points) and of the white matter involvement (0–6 points) were included. The deep grey matter involvement was the sum of thalamus, putamen, PLIC and four-column sign subscores. White matter involvement included oedema, subcortical white matter and periventricular white matter subscores.

**Fig. 2:**
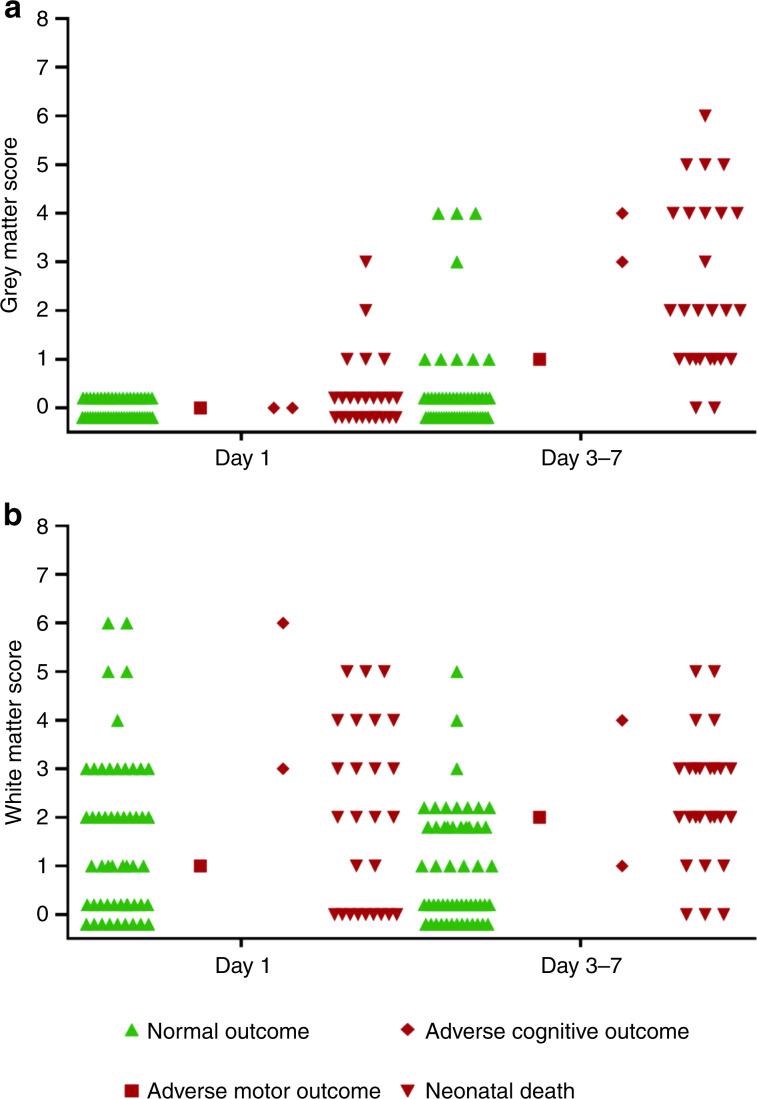
Cerebral ultrasound, outcome and HIE: subscores. Grey matter subscores (**a**) and white matter subscores (**b**) of cohort I on day 1 and day 3−7, categorised for outcome.

The grey and white matter subscores of cohort I on both days are shown in Fig. 2 per outcome group.

The cumulative score of the white matter (OR = 2.6, 95% CI 1.5–4.7) and of the deep grey matter involvement (OR = 2.7, 95% CI 1.7–4.4) between days 3 and 7 after birth were included in the final scoring system. The RI was not significant in multivariable logistic regression. The probability of an adverse outcome at the age of 2 years could be calculated using the following formula: 1/(1 + e^−(−3.385 + 0.960 × white matter + 0.995 × deep grey matter)^). The CUS scoring system performed well in cohort I (AUC = 0.90; 95% CI 0.83–0.98). Table 4 shows the performance per cut-off value.

**Table 4:** Performance of the model in cohort I and cohort II.

Cut-off value^a^	≥3	≥4	≥5	≥6	≥7
Cohort I					
Sensitivity	93% (76–99)	79% (60–91)	45% (27–64)	28% (13–47)	17% (7–36)
Specificity	86% (74–94)	88% (76–95)	92% (81–96)	98% (88–99)	100% (91–100)
PPV	79% (62–91)	79% (60–91)	76% (50–92)	89% (59–99)	100% (46–100)
NPV	96% (84–99)	88% (76–95)	75% (62–85)	71% (59–81)	68% (57–78)
Cohort II					
Sensitivity	75% (47–92)	69% (41–88)	63% (36–84)	56% (31–79)	44% (21–69)
Specificity	94% (68–100)	94% (68–100)	100% (76–100)	100% (76–100)	100% (76–100)
PPV	92% (62–100)	92% (60–100)	100% (66–100)	100% (63–100)	100% (56–100)
NPV	79% (54–93)	75% (51–90)	73% (50–88)	70% (47–86)	64% (43–81)

*PPV* positive predictive value, *NPV* negative predictive value.

^a^A cut-off value of ≥3 means that an ultrasound score of 3 or more is defined as abnormal.

### Validation: inter-observer variability

Table 5 shows the agreement between the observers in cohort I. There is a moderate inter-observer agreement between all three observers.

**Table 5:** Agreement between the observers in cohort I.

	Observer 1 vs. 2	Observer 1 vs. 3	Observer 2 vs. 3
Spearman’s rho	0.74 (*p* = 0.001)	0.64 (*p* < 0.001)	0.72 (*p* = 0.001)

### Validation of the scoring system in cohort II

The predictive values of the scoring system in cohort II are shown in Table 4. The AUC was 0.89 (95% CI 0.77–1.00).

To exclude the effect of the hospital on outcome, logistic regression was performed with the total CUS score, the hospital and their interaction term included in the analysis. The CUS score was significantly associated with adverse outcome (OR = 2.5; 95% CI 1.8–3.4), the hospital and their interaction term were not.

### Validation: correlation with MRI and histology

**Fig. 3:**
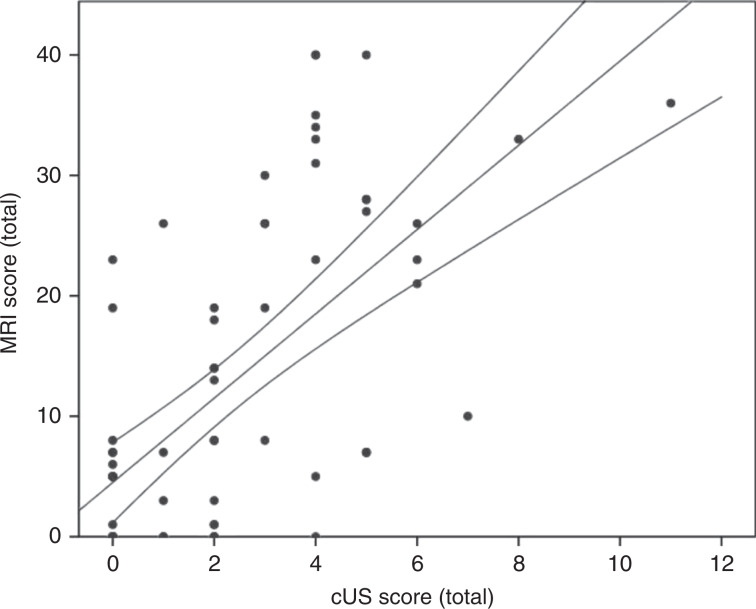
The correlation between the MRI and CUS scoring system in cohort I.

There was a moderate correlation between the CUS and MRI scoring system in cohort I (Spearman’s rho = 0.67; *p* < 0.001; Fig. 3). In the six most severely affected infants, an MRI was not feasible because the infants were clinically too unstable.

Of the 26 infants in cohort I that died during the neonatal period, 17 infants (65%) underwent postmortem examination. In all these 17 infants, CUS abnormalities were confirmed with histology. The histological damage was more extensive than diagnosed with CUS.

## Discussion

We developed a scoring system to structurally score CUS abnormalities in (near)-term infants with HIE. The scoring system was associated with neurodevelopmental outcome and includes composite scores of white matter and deep grey matter involvement, both of which contain multiple separate items. This scoring system was developed based on the CUS between days 3 and 7 after birth; the CUS on day 1 after birth was not predictive of adverse outcome. The CUS scoring system was validated in another cohort and the performance was relatively good.

To the best of our knowledge, this is the first CUS scoring system using a validated composite score to predict an adverse outcome. Currently, three CUS scoring systems for infants with HIE are available. A CUS scoring system for asphyxiated infants in Uganda has recently been reported.^17^ This scoring system was used to identify early HIE-related brain damage but did not provide predictive values.^17^ Two other scoring systems were developed to score patterns of brain injury in HIE. Leijser et al.^26^ scored combinations of white and grey matter involvement and compared CUS and MRI. The other CUS scoring system by Swarte et al.^28^ defined six different patterns, for example, the combination of deep grey matter involvement and extensive cortical involvement. These scoring systems did not allow different items to be scored within the categories separately. For example, when describing deep grey matter involvement, this may imply that the left and right thalamus are affected but the basal ganglia are not, while it is of importance to distinguish between just thalamic involvement and thalamic and basal ganglia involvement. Furthermore, they combined white and grey matter involvement, even though different types of brain injury might lead to different outcomes.^5^ For these reasons, we developed a CUS scoring system based on composite scores, which might be easier to use in clinical practice. The composite scores for deep grey matter and white matter involvement had to be summed because of multicollinearity. However, it remains possible to score the different items separately and to make a distinction between white matter and deep grey matter involvement. Additionally, this is the first CUS scoring system in HIE that is validated in another cohort.

As expected, all scored items on day 3−7 in the univariate analysis were significant predictors of adverse outcome. These items have all been described as asphyxia-related brain injury.^19,23,25,26,30–32^ Asphyxia-related brain injury is more common in HIE, but as many as 34.2% of controls also showed periventricular hyperechogenicity and 9.2% slit-like ventricles shortly after birth.^22^ Further, Eken et al. correlated hyperechogenicity on CUS with histological findings: hyperechogenicity of the thalamus occurred within 72 h after birth on CUS (sensitivity 100%, specificity 83.3% of CUS compared to histology), hyperechogenicity of the periventricular white matter within 24 h (sensitivity 100%, specificity 83.3%) and hyperechogenicity of the cortex within 72 h (sensitivity 76.9%, specificity 100%). Additional lesions, not identified by CUS, were found in the brainstem, hippocampus and cerebellum with histology.^21^ These three items were not included in our CUS scoring system. The “four-column sign” and visibility of the PLIC were included as separate items because in some infants the PLIC was visible, but there was no clear “four-column sign”.

The CUS conducted on day 1 after birth was not predictive of outcome in this study, which is in agreement with previous studies.^17,22^ As mentioned above, it takes 24−72 h before brain injury becomes visible as hyperechogenicity on CUS, unless the onset of the injurious process is of antenatal onset.^21^ Consequently, CUS within 6 h after birth had in a previous study a low sensitivity of 42.1% and specificity of 60%.^23^ Nevertheless, CUS on day 1 is recommended to identify antenatally acquired pathology.^5,10,11,32^ We indeed found antenatally acquired pathology in 14 of the 83 infants in cohort I. Most of the antenatally acquired lesions, i.e. germinal layer cysts, did not influence outcome and can also be found in controls.^17^ However, in one infant a porencephalic cyst was found that led to a mildly asymmetrical motor outcome.

It was of interest to see that the RI was not associated with adverse outcome in multivariable logistic regression analyses. The RI was highly predictive in previous studies with non-cooled infants, but appears to be less predictive in cooled infants, as reported previously.^20,21,31,33^ Especially the positive predictive value has decreased, so the observed outcome is better than the expected outcome based on an abnormal RI. It has been hypothesised that hypothermia has a direct effect on the cerebral vessels or that hypothermia leads to a better neurodevelopmental outcome but does not lead to a normalisation of the RI.^33^

The correlations between MRI and CUS in a study of Leijser et al. were stronger than in our cohort (0.83 versus 0.67).^26^ This might be explained by the fact that they used exactly the same scoring systems for MRI and CUS and in our cohort the scoring systems differed i.e. the cerebellum was included in the MRI scoring system but not in the CUS scoring system. In cohort I there was a moderate correlation between MRI and CUS, but predictive values for MRI in the study of Weeke et al. were higher than for CUS in our study.^13^ The most severely affected infants were too ill to undergo an MRI. We speculate that these would have shown severe MRI abnormalities, thereby improving the overall association between CUS and MRI. The performance of the model was good in both cohorts for both the cut-off value of ≥3 which can potentially be used if the number of false negatives should be as low as possible (i.e. for decisions about additional future neuroprotective strategies) and for higher cut-off values that can be preferable if the number of false positives should be as low as possible (i.e. for considering redirection of care in combination with neurophysiology and clinical features if MRI is not possible). The interrater variability was moderate between all observers implying that the interrater agreement among different hospitals and observers would also be moderate. In a prospective cohort, in which observers can be trained and the quality of CUS can be guaranteed, interrater variability can be further improved.

Our study has several limitations. The first limitation is the retrospective design; no images were routinely taken based on a standard scan protocol. This resulted in poor quality of the images due to non-optimal settings in some cases, the absence of certain anatomical structures on the available images, the absence of CUS on certain days and the absence of follow-up data. As a consequence, a relatively large number of infants had to be excluded. Secondly, neonatal death due to redirection of care was defined as an adverse outcome; the decision was based on a combination of clinical findings, neurophysiology and neuroimaging findings. Even so, we cannot exclude that this leads to some bias because it is not certain that these infants would have experienced problems later in life. However, in cohort I 17 of the 26 infants that died had a postmortem examination and in all infants CUS findings were confirmed. Histopathology showed more extensive damage than CUS, which has also been described for MRI.^34^ Thirdly, because of the relatively small sample size and the low incidence of adverse motor or cognitive outcome, our study was not powered enough to perform sub-analyses for the different outcome parameters. Finally, the distinction between the categories “normal to mild” and “moderate” hyperechogenicity remains subjective. Severe hyperechogenicity is easier to distinguish from the other categories. However, this is a reflection of clinical practice and with this scoring system we finally have a method that supports the clinician in their daily routine. Furthermore, this scoring system provides a tool for prospective clinical trials. Probably, the predictive value of the CUS scoring system will further improve when researchers and clinicians will focus even more on the quality using standard scan protocols in prospective studies.

In summary, this novel CUS scoring system provides a tool to structurally assess brain injury and predict outcome in HIE if MRI is not feasible or available. It is an easy tool to use in clinical practice and is the first validated CUS scoring system in HIE. In the future, this CUS scoring system should be tested prospectively in infants with HIE.

## Data Availability

The datasets generated and analysed during the current study are available from the corresponding author on reasonable request.
